# Integrating mental health into chronic care in South Africa: the development of a district mental healthcare plan

**DOI:** 10.1192/bjp.bp.114.153726

**Published:** 2016-01

**Authors:** Inge Petersen, Lara Fairall, Arvin Bhana, Tasneem Kathree, One Selohilwe, Carrie Brooke-Sumner, Gill Faris, Erica Breuer, Nomvula Sibanyoni, Crick Lund, Vikram Patel

**Affiliations:** **Inge Petersen**, PhD, School of Applied Human Sciences, University of KwaZulu-Natal, Durban, South Africa; **Lara Fairall**, PhD, Knowledge Translation Unit, University of Cape Town, Cape Town, South Africa; **Arvin Bhana**, PhD, **Tasneem Kathree**, MSocSci, **One Selohilwe**, MSocSci, **Carrie Brooke-Sumner**, MSc, School of Applied Human Sciences, University of KwaZulu-Natal, Durban, South Africa; **Gill Faris**, MPhil, Knowledge Translation Unit, University of Cape Town, Cape Town, South Africa; **Erica Breuer**, MPH, Alan J Flisher Centre for Public Mental Health, Department of Psychiatry and Mental Health, University of Cape Town, Cape Town, South Africa; **Nomvula Sibanyoni**, MSocSci, National Department of Health, Pretoria, South Africa; **Crick Lund**, PhD, Alan J Flisher Centre for Public Mental Health, Department of Psychiatry and Mental Health, University of Cape Town, Cape Town, South Africa, and Centre for Global Mental Health, Institute of Psychiatry, Psychology and Neuroscience, King's College London, UK; **Vikram Patel**, PhD, Centre for Global Mental Health, London School of Hygiene and Tropical Medicine, London, UK and Centre for Mental Health, the Public Health Foundation of India, India

## Abstract

**Background**

In South Africa, the escalating prevalence of chronic illness and its high comorbidity with mental disorders bring to the fore the need for integrating mental health into chronic care at district level.

**Aims**

To develop a district mental healthcare plan (MHCP) in South Africa that integrates mental healthcare for depression, alcohol use disorders and schizophrenia into chronic care.

**Method**

Mixed methods using a situation analysis, qualitative key informant interviews, theory of change workshops and piloting of the plan in one health facility informed the development of the MHCP.

**Results**

Collaborative care packages for the three conditions were developed to enable integration at the organisational, facility and community levels, supported by a human resource mix and implementation tools. Potential barriers to the feasibility of implementation at scale were identified.

**Conclusions**

The plan leverages resources and systems availed by the emerging chronic care service delivery platform for the integration of mental health. This strengthens the potential for future scale up.

South Africa has a 12-month prevalence estimate of 16.5% for common mental disorders (anxiety, mood and substance use disorders),^1^ with almost a third (30.3%) of the population having experienced a common mental disorder in their lifetime.^2^ These estimates are relatively high when compared with international prevalence estimates of the WHO (World Health Organization) World Mental Health surveys.^3^ As is the case internationally,^4,5^ the treatment gap in South Africa is also high, with only one in four people with a common mental disorder receiving treatment of any kind. For those with psychotic disorders, although identification and access to treatment is better, there are insufficient resources at community level for promotion of recovery.^6,7^ The aim of the formative phase of the PRogramme for Improving Mental health carE (PRIME) in South Africa was to (a) develop a mental healthcare plan (MHCP), customised to local conditions at district level that provides acceptable and feasible collaborative care packages for depression, alcohol use disorders and schizophrenia and can be integrated into existing service delivery platforms; (b) identify the human resource mix to deliver the MHCP and develop implementation tools to support and facilitate scale up of the packages; and (c) identify potential barriers to implementation at scale. The reason for the focus on these conditions is their relatively high burden of disease and disability and evidence of cost-effective interventions for their treatment.^8^

## Method

### Context

Although South Africa is an upper-middle-income country, there are large disparities in wealth and access to resources. Within the health sector, these disparities are reflected in inequities between private and public health provision. Private healthcare, funded through private health insurance and out of pocket payments, serves approximately 16% of the population, compared with about 84% served by public healthcare. Yet gross domestic product spend on each is similar (4.1% and 4.2% respectively).^9^ To redress these inequities, South Africa is phasing in (over 14 years) a national health insurance system, to ensure universal access to appropriate, efficient and high-quality health services.^9^ The introduction of national health insurance involves an overhaul of services as well as systems to support service delivery. Notably, at the district-service level is re-engineering of primary healthcare. This includes the establishment of district specialist clinical teams to provide support to ward-based primary healthcare teams. The latter comprise primary healthcare staff at fixed primary healthcare facilities as well as community outreach teams consisting of a professional nurse and community health workers.^10^

Embedded within the national health insurance system, is the introduction of integrated chronic disease management (ICDM) to meet the needs of the rising number of patients with multiple chronic diseases associated with the roll-out of antiretroviral therapy and a burgeoning non-communicable diseases epidemic.^11^ At the facility level, the ICDM aims to strengthen the quality of care for chronic conditions through: (a) consolidating services for all chronic care patients, including those with communicable and non-communicable diseases, into a single delivery point; and (b) strengthening clinical decision support through the adoption of an integrated set of nurse-led clinical guidelines developed for the identification and management of multiple chronic diseases, called Primary Care 101 (PC101).^12^ At community level, community outreach teams support clinically stable patients in order to promote self-management. At a population level, health promotion and population screening are envisaged to promote an informed and activated population.^13^ Within this context, mental health is gaining ground as a public health priority. It is increasingly understood to be integral to the delivery of chronic care given that depression and alcohol misuse compromise prevention efforts as well as adherence to treatment.^14–16^

Legislative and policy developments specific to mental health include the introduction of a new Mental Health Care Act (No 17 of 2002) in 2004, as well as a new national mental health policy framework and strategic plan (2013–2020). Both promote decentralised and integrated care through task sharing. A noteworthy development that will enable implementation is the introduction of specialist district mental health teams expected to play a public mental health role.

Against this background, the South African national Department of Health advised that PRIME in South Africa focus on integrating mental health services for depression and alcohol use disorders into the ICDM service delivery platform given departmental priorities to reduce mortality as a result of chronic conditions, including HIV/AIDS. In light of service gaps in community-based psychosocial rehabilitation for patients with schizophrenia,^6^ this became a further focus of PRIME in South Africa. Ethical approval for the formative phase of PRIME, including the pilot study, was obtained from the University of KwaZulu-Natal Ethics Committee (HSS/0880/011 and BE 317/13) and the Human Research Ethics Committee of the Faculty of Health Sciences, University of Cape Town (REC Ref: 412/2011). All participants involved in semi-structured interviews consented to participating in the studies using approved informed consent procedures.

### Study site

The Dr Kenneth Kaunda District (DKK) in the North West Province was chosen as the study site by the Department of Health as it is one of three districts where ICDM is being piloted in the country, and is a pilot site for national health insurance and the re-engineering of primary heathcare. DKK is in the southern part of the North West Province, which is located immediately west of the populous Gauteng province. Please see online Fig. DS1 for a map of South Africa with the location of DKK highlighted. DKK comprises four subdistricts, with a population of approximately 796 823, the majority of whom (90%) are urban. The main economic activities are mining and agriculture. Public health facilities include regional hospitals, primary healthcare facilities and one specialist in-patient mental health facility (details are contained in online Table DS1). Private healthcare facilities are also available but were not the focus of study given the emphasis on integrating mental health into the public service ICDM service delivery platform.

### Research approach

A mixed methods approach to the formative phase was used: a situational analysis; theory of change (ToC) workshops; qualitative interviews with service managers, service providers, patients and carers; and piloting of the preliminary MHCP in one clinic.

#### Situational analysis

A generic situational analysis tool developed for use across all PRIME country sites (downloadable from http://www.prime.uct.ac.za/images/prime/PRIME_Final_Situational_analysis_Tool.pdf) was adapted for the South African site. This tool required that information be gathered on a range of contextual issues such as burden of disease; mental health policies, plans and legislation; estimated treatment coverage for mental and neurological disorders; available resources and mental health information systems. Data were collected via secondary data sources at national and district level. The results of this situational analysis are reported elsewhere.^17^

#### Theory of change workshops

Participatory theory of change (ToC) workshops were held with key stakeholders including service managers, service providers and patients to develop a MHCP for the district. ToC provides a useful framework for guiding the development and ownership of complex health interventions such as an MHCP, starting with the intended impact and working backwards to identify the outcomes to achieve the impact, and the inputs and processes needed to achieve these outcomes.^18^ A more detailed account of the process is provided by Breuer *et al*.^19^

Discussion in the ToC workshops centred on how key outcomes identified in the cross-country ToC map^20^ could be achieved in the district for each priority condition. At the organisational level the key cross-country packages comprised engagement and mobilisation, programme implementation and management and capacity building. At the facility level, they comprised mental health literacy, identification and diagnosis, drug treatment, psychosocial interventions and continuing care. The community level included mental health literacy, detection and referral, adherence support, rehabilitation and mobilisation.

In total three ToC workshops were held over a period of 6 months from March to August 2012 to develop the initial plan. One workshop involved service providers and managers from facility level to the national level (*n* = 26); one was with community-based service providers and patients (*n* = 21); and one combined the above two groups (*n* = 31), which merged and refined the ToC maps into a final one, drawing on information from the formative qualitative interviews as well.

#### Qualitative formative interviews

Formative qualitative interviews were undertaken with key stakeholders to maximise the social and cultural fit of the emergent MHCP, including an acceptable human resource mix and associated tools to support implementation. All interviews were audiotaped. In total there were 79 individual interviews with patients (*n* = 70) and caregivers (*n* = 9); 47 with service providers made up of 25 individuals and 4 focus groups (*n* = 22). All interviews were audiotaped. The interviews were translated where necessary with back translation checks applied and transcribed verbatim. The transcripts were analysed with the help of NVivo 10 qualitative data analysis software, using framework analysis by stakeholder group.^21^ This involved a number of steps including: (a) reading and re-reading the transcripts; (b) the development of a coding framework based on the interview questions; (c) coding of the data, with emergent themes being added to the coding framework during this coding process; (d) summarising the responses from the respondents across each theme; and (e) interpreting the final themes in light of what they suggested for service planning and interventions. The results of the formative interviews are reported by stakeholder group in detail in a number of published articles.^22–24^

#### Draft collaborative care packages

**Fig. 1:**
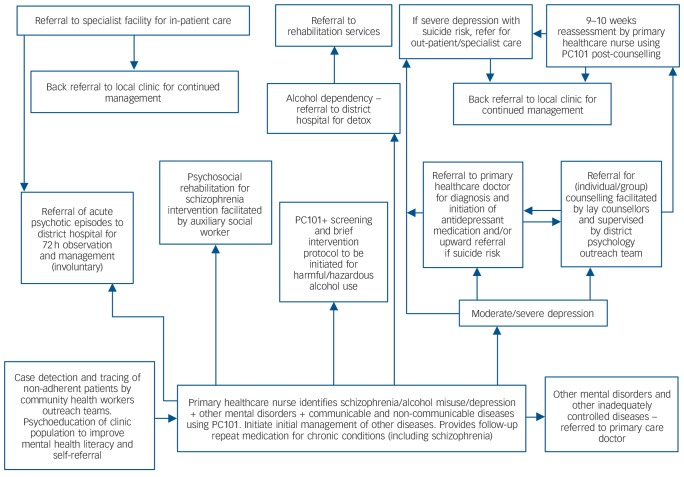
Collaborative care packages for schizophrenia, alcohol misuse and depression. PC101, Primary Care 101.

The situational analysis, ToC workshops and formative interviews informed the development of preliminary collaborative care packages depicted in Fig. 1. At the community level, improved identification for all the disorders was to be achieved through the second phase of the Department of Health community health worker outreach team training programme, which includes training in identification and referral of people with mental disorders. These community health worker outreach teams are also responsible for tracing all patients with chronic care needs who default on treatment. At primary healthcare facility level, improved identification and diagnosis of comorbid depression, alcohol use disorders and schizophrenia by primary healthcare nurses and doctors was to be achieved through strengthening the mental health guidelines of PC101 to ensure alignment with the WHO's Mental Health Gap Action Programme (mhGAP) algorithms (subsequently referred to as PC101+); adding a psychoeducational page for the ‘stressed/ miserable patient’; and adding additional mental health cases to the PC101 training.

For the depression package, patients with mild symptoms were to be provided with basic psychoeducation using the ‘stressed/miserable patient’ page; patients with moderate to severe depression were to be referred to a primary healthcare doctor for initiation of antidepressant medication and/or to a facility-based lay counsellor for initiation of individual or group counselling using the PRIME South Africa depression counsellor guidelines adapted from a local intervention using evidence-based approaches of interpersonal therapy and cognitive–behavioural techniques.^25,26^ (The term ‘lay counsellor’ is used in the South African context to differentiate this group from other counsellors who have formal qualifications in counselling. ‘Lay counsellors’ typically receive minimal training in counselling to provide a limited and prescribed service.) After 8 weeks of counselling (the optimum number used in an effective collaborative task sharing intervention for depression in India^27^) patients were to be reassessed by a primary healthcare nurse using PC101+ and referred onwards for specialist care if necessary. Patients on medication were expected to be monitored on a monthly basis when they come to collect their repeat medication.

For the alcohol misuse package, primary healthcare nurses/doctors were expected to provide psychoeducation on what constitutes acceptable levels and patterns of alcohol use for patients with harmful or hazardous drinking using guidelines introduced into PC101+. This has been shown to be an effective strategy for reducing alcohol consumption globally.^28^ mhGAP guidelines for the management of alcohol withdrawal at primary healthcare level were included in PC101+, with onward referral to the local district hospital for detoxification and specialised rehabilitation services.

In the case of the schizophrenia package, according to the Mental Health Care Act,^29^ patients with acute conditions are admitted to the nearest designated district hospital that provides 72 h observation and stabilisation. If necessary they are then referred onwards to a specialist mental health facility for diagnosis and initiation of treatment. On being discharged, patients are expected to attend their nearest primary healthcare facility as a chronic care patient for ongoing medication. In the PRIME collaborative care package, these stabilised patients were to be referred to community-based psychosocial rehabilitation groups facilitated by auxiliary social workers from the Department of Social Development/local Mental Health Society (a Mental Health non-governmental organisation (NGO)). Recent evidence attests to the effectiveness of community-based task sharing approaches for reducing disability and symptoms of schizophrenia in India.^30^

#### Pilot study

These collaborative care packages were piloted from August to November 2013 in one clinic in DKK identified by the district management team. The identified clinic is situated in a peri-urban area of the district and saw an average of 3000 patients per month in 2013. Implementation involved a series of training workshops for personnel who formed part of the human resource mix required to deliver the packages of care.

Evaluation and monitoring of the implementation of the pilot involved monitoring implementation via implementation logs as well as process evaluation interviews. Process evaluation interviews were conducted at 3-month follow-up with service providers trained to deliver the intervention packages and patients who received the interventions. The purpose of the qualitative process evaluation interviews was to gain an understanding of individual's experience of delivering and receiving the interventions as well as bottlenecks that emerged and reasons for these. Participants included primary healthcare nurses (*n* = 4), lay counsellors (*n* = 4), auxiliary social workers (*n* = 2), patients who received counselling for depression (*n* = 6), patients who attended psychosocial rehabilitation groups (*n* = 6) and caregivers of patients attending psychosocial rehabilitation groups (*n* = 4). The interviews were translated and transcribed verbatim. Framework analysis, described previously, was used to analyse the data.

## Results

For depression, over a 3-month period, only 15 patients were identified by the primary healthcare nurses and referred to the counsellors for depression. Two-thirds of patients referred (*n* = 10) presented for their counselling appointment and of these, 7 attended follow-up counselling sessions following the initial session. There were no recorded referrals to the primary healthcare doctor. No patients with alcohol use disorders were identified. For schizophrenia, 19 patients who had been down-referred from the psychiatric hospital to receive their follow-up medication from the clinic were identified from the clinic records. Of these, only nine were attending the clinic for their follow-up medication of which six eventually attended the psychosocial rehabilitation group on a regular basis, with one dropping out. Only one caregiver attended the caregiver sessions.

The bottlenecks identified by the pilot study thus included a paucity of referrals by primary healthcare nurses to the lay counsellors for depression counselling; minimal identification of alcohol use disorders; poor follow-up of counselling referrals made by counsellors; a high default rate of patients receiving follow-up medications for mental illness at the primary healthcare clinic, which limited the number of patients who could be accessed for psychosocial rehabilitation groups; and poor uptake of the psychosocial rehabilitation intervention by caregivers of patients with schizophrenia.

Reasons for these bottlenecks that emerged from the qualitative process interviews included the following.

- On the part of patients/caregivers, poor mental health literacy was a barrier to help-seeking for depression; defensiveness in divulging alcohol consumption was a barrier for identification of alcohol use disorders.
- On the part of nurses, barriers to identification and/or referral rates of depression and alcohol use disorders was a result of low self-confidence in ability to diagnose common mental disorders; unattended personal issues; focusing on underlying social problems and referral to social workers without attending to the presenting mental disorders; and lack of confidence in lay counsellor abilities.
- On the part of counsellors, in addition to unattended personal issues; marginalised status and unclear roles; low confidence; and poor suitability of some counsellors emerged as being reasons for poor follow-up of patients referred to them for counselling.
- Structural and organisational challenges that impeded identification and/or referral of depression or alcohol use disorders by nurses included high patient loads and space constraints that limited consultation.
- Space constraints also emerged as limiting confidentiality of counselling.
- With regard to the psychosocial rehabilitation group intervention, a high default rate and poor tracing of individuals who defaulted limited the number of patients who could be referred to the groups by the primary healthcare nurses.

A feedback meeting was held with key stakeholders including district managers, facility managers, service providers and patients in February 2014 to discuss the results of the pilot study, collectively identify strategies to overcome emergent bottlenecks and adapt the MHCP following the approach used by the International Health Institute for health systems strengthening. This approach uses a continuous quality improvement approach to bring healthcare facilities together at regular intervals to jointly identify and solve bottlenecks that emerge in the improvement of health system innovations.^31^

To aid roll-out, the implementation tools have been strengthened to include an implementation toolkit comprising implementation guidelines for district and facility managers, providing a step-by-step guide for the implementation and integration of mental health services at facility level. This toolkit also incorporates change management to orientate managers and service providers to the ethos and organisational needs of the collaborative chronic care, including providing more containing leadership and stress management; establishing targets for identification and treatment of priority mental disorders; and role clarification of the different team members where the primary healthcare nurse is designated as the case manager; lay counsellors designated to provide counselling for depression; auxiliary social workers are designated to provide psychosocial rehabilitation for patients with schizophrenia; and community health workers' role in following up patients who have defaulted on treatment and/or psychosocial intervention is emphasised. The need to strengthen the existing employee wellness programme at an organisational level was also highlighted.

The piloting process also informed how the guidelines and training materials in the toolbox needed to be strengthened. Noteworthy is modification of the alcohol use disorders guidelines to take into account patients' defensiveness in acknowledging high levels of alcohol use as patients with chronic care needs are often told to abstain from drinking alcohol; the introduction of waiting room educational talks in addition to information leaflets to improve mental health literacy; and the strengthening of referral documentation and information collected on the priority conditions. All the training materials, guidelines and resources comprise an ‘implementation toolbox’ for integrating mental healthcare at district level in South Africa (see online Table DS2 for a comprehensive list). A summary of the findings of the piloting process and how they informed modifications to the MHCP and adaptation of the implementation tools is provided in Table 1.

**Table 1:** Bottlenecks identified in pilot study, reasons and modifications to the mental healthcare plan (MHCP) and implementation tools

Bottleneck and reasons	Modifications to MHCP and implementation tools
*Paucity of referrals for depression to lay counsellors by primary healthcare* *nurses and minimal identification of alcohol use disorders*	
Organisational	
High patient loads	Change management for district/facility managers and service providers
Limited time and space for consultation needed to identify symptoms	Inclusion of depression on chronic care form
Weak information system for mental health	Facility target for identification of depression/alcohol use disorders
Primary healthcare providers	
Biomedical orientation	Alcohol use disorders PC101+ guidelines strengthened
Unattended personal issues	Strengthened employee assistance programme
Psychiatric stigma	
Perception of depression/alcohol use disorders as a social problem requiring referral to a social worker	
Patients	
Low mental health literacy	Waiting room educational talks
Defensiveness in divulging alcohol consumption	Information leaflets

*Low follow-up of counselling referrals by counsellors*	
Organisational	
Unclear role clarification of lay counsellor roles	Inclusion of primary healthcare nurse as case managers
Marginalised status	
Primary healthcare providers	
Low self-esteem	Role clarification of primary healthcare nurses and lay counsellors in PC101+ training
Lay counsellors	
Unattended personal issues	Role clarification and stress management in lay counsellor training
Poor suitability to counselling role	Selection of dedicated lay counsellors
Patients	
Low mental health literacy	Waiting room educational talks
	Information leaflets

*High rate of defaulting on follow-up medication*	
Organisational	
ICDM (no dedicated queue or nurse for psychiatric patients)	Strengthened role of community health workers in tracking patients with mental disorders
Poor tracking of defaulters	
Primary healthcare providers	
Psychiatric stigma	Strengthened orientation to mental health in PC101 +
Poor understanding of severe mental illness	
Patients	
Low mental health literacy	Psychosocial rehabilitation intervention
	Information leaflets

*Low uptake of psychosocial rehabilitation intervention by caregivers*	
Organisational	
Poor community outreach to families	Strengthened role of community outreach to provide psychoeducation
Primary healthcare providers	
Poor understanding of the need to provide psychoeducation to families	Strengthened PC101+ training
Caregivers	
Low mental health literacy	Focused engagement of caregivers by community outreach teams prior to programme
Psychiatric stigma	

PC101, Primary Care 101; ICDM, integrated chronic disease management.

The final MHCP is depicted in Tables 2, 3 and 4. It comprises core intervention packages at the organisational (Table 2), facility (Table 3) and community (Table 4) levels identified through the ToC process to achieve the identified outcomes along the continuum of care (see Method), with the addition of employee wellness at the organisational level. These packages comprise the human resources required to deliver each package as well as resources/mechanisms to aid implementation.

**Table 2:** Core intervention packages at the organisational level

	Engagement and mobilisation	Programme implementation	Programme management	Capacity building	Employee wellness
Resources/mechanisms	PRIME South Africa implementation toolkit including change management	PRIME South Africa implementation toolbox comprising step-by-step guide for implementing the MHCP as well as implementation tools	District management team meetings Mental health information system	Trainer of trainers interactive tools for PC101+ Counselling guidelines Psychosocial rehabilitation guidelines Supervision guidelines	Employee assistance programme Providers/managers: change management includes stress management

Human resources	District and facility managers	District and facility managers, district trainers	Provider/manager: district managers Information managers	Provider/manager: district trainers	All district and facility managers and service providers

PRIME, PRogramme for Improving Mental health carE; PC101+, Primary Care 101+.

**Table 3:** Core intervention packages at the facility level

	Mental health literacy	Detection/diagnosis and referral	Drug interventions	Psychosocial interventions	Continuing care
Depression					
Resources/mechanisms	Waiting room talk and pamphlets PC101+ includes orientation to importance of mental health and anti-stigma	PC101+ Collaborative care model	PC101+ Essential drug list	PC101+ includes psychoeducation for ’stressed/miserable client’ PRIME South Africa depression counselling guidelines	Stepped up collaborative care model Referral documentation
Human resources	Primary healthcare nurses, doctors, health promoters, non-professional counsellors	Primary healthcare nurses/doctors	Primary healthcare nurses/doctors	Primary healthcare nurses/doctors Lay counsellors	Primary healthcare nurses/doctors

Alcohol use disorders					
Resources/mechanisms	Waiting room talk and pamphlets PC101+ includes orientation to importance of mental health and anti-stigma	PC101+ Collaborative care model	PC101+ Essential drug list	PC101+ screening and brief intervention	Stepped up collaborative care model Referral documentation
Human resources	Primary healthcare nurses, doctors, health promoters, non-professional counsellors	Primary healthcare nurses/doctors	Primary healthcare nurses/doctors	Primary healthcare nurses/doctors	Primary healthcare nurses/doctors

Schizophrenia					
Resources/mechanisms	Waiting room talk and pamphlets PC101+ includes orientation to importance of mental health and anti-stigma	PC101+ Collaborative care model	PC101+ Essential drug list	PC101+ includes psychoeducation on schizophrenia Psychosocial rehabilitation groups	Stepped up collaborative care model Referral documentation
Human resources	Primary healthcare nurses, doctors, health promoters, lay counsellors	Primary healthcare nurses/doctors	Primary healthcare nurses/doctors	Primary healthcare nurses/doctors Auxiliary social workers	Primary healthcare nurses/doctors

PRIME, PRogramme for Improving Mental health carE; PC101+, Primary Care 101+.

**Table 4:** Core intervention packages at the community level

	Mental health literacy	Detection and referral	Outreach adherence support	Rehabilitation	User mobilisation
Depression					
Resources/mechanisms	2nd phase Department of Health community health worker training programme	2nd phase Department of Health community health worker training programme	Proactive tracing of patients that are non-adherent		
Human resources	Community health workers	Community health workers	Community health workers		

Alcohol use disorders					
Resources/mechanisms	2nd phase Department of Health community health worker training programme	2nd phase Department of Health community health worker training programme	Proactive tracing of patients that are non-adherent	Department of Social Development Rehabilitation centres	
Human resources	Community health workers	Community health workers	Community health workers		

Schizophrenia					
Resources/mechanisms	2nd phase Department of Health community health worker training programme	2nd phase Department of Health community health worker training programme	Proactive tracing of patients that are non-adherent	PRIME South Africa psychosocial rehabilitation guidelines	User engagement session added to PRIME South Africa psychosocial rehabilitation guidelines
	PRIME South Africa psychosocial rehabilitation guidelines				
Human resources	Community health workers Auxiliary social workers	Community health workers	Community health workers	Auxiliary social workers	Auxiliary social workers Health promoters

PRIME, PRogramme for Improving Mental health carE.

Based on the collaborative care models for the different disorders, the implications for the human resource mix, their roles and responsibilities and associated resources/mechanisms required to implement the plan at scale are depicted in Table 5. Identification of the human resource mix is important to inform core competencies and curricula of training programmes; and the role of regulatory and accreditation bodies in ensuring the production of a workforce equipped with the necessary competencies for scale up.

**Table 5:** Human resource mix for PRogramme for Improving Mental health carE (PRIME) South Africa mental healthcare plan

Human resource	Function/Services	Resource/mechanism/tool
District/subdistrict management tier		
District management team (chief director; director: primary healthcare services; director: hospital services; manager: subdistrict director: primary healthcare services; district manager: training)	Management of programmes for example maternal health, HAST (HIV/AIDS/sexually transmitted infections/tuberculosis) Ensure collaboration with other services Ensure capacity building	District management team meetings Training programmes
Subdistrict team (directors: primary healthcare services; community health services; coordinators: chronic care/ mental health; counselling)	Management of all programmes including chronic care	Subdistrict management team meetings PRIME implementation toolkit including change management workshop Social cluster meetings to promote intersectoral collaboration
Information officer	Plan, manage and monitor the mental health components of the district information system	Existing MHIS Provincial indicator data-set, district health information system Patient register in terms of Section 39 of the Regulations to the Mental Health Care Act No 17 of 2002 for health establishments that render mental health services
District/subdistrict pharmacist	Ensuring sufficient stock of psychotropic medication Dispensing of medication and distribution to primary healthcare Packaging of psychotropic medication for patient	Standard treatment guidelines and essential drug list – hospital level adults 2006 Standard treatment guidelines and essential medicines list for primary healthcare 2008

Tertiary hospital tier		
Psychiatrist	Assessment, diagnosis and holistic treatment (including psychiatric treatment) of in-patients/ out-patients Consultation-liaison service for primary healthcare doctors and other mental health specialist (outreach and support) Training and supervision of registrars	Hospital protocol Standard treatment guidelines and essential drug list – hospital level adults 2006
Clinical psychologist	Assessment, diagnosis and psychological treatments for in-patients and out-patients Psychoeducation of patients/families Training of intern psychologists	Hospital protocol
Psychiatric nurse	In-patient care/out-patient nursing care Psychosocial rehabilitation Psychoeducation of families	Hospital protocol
Clinical social worker	Family assessment and psychoeducation Psychoeducation of families Placement in alternative accommodation and/or sheltered workshops on discharge Assist psychiatric patients with applications for disability grants	Hospital protocol
Occupational therapist	Assessment and psychosocial rehabilitation	Hospital protocol
Pharmacist	Orders and dispenses medication	Hospital protocol and essential drug list
Dietician	Prepares menus	Hospital protocol
Neurologist (part time)	Assesses electroencephalograms	Hospital protocol
Information officer	Capture and manage patient records	District information system

District hospital tier		
Clinical psychologist	Assessment, diagnosis and psychological treatments for in-patients (emergency admissions, 72 h observation of involuntary patients, short-term acute in-patient care and upward referral to tertiary specialist services Psychological referral service for complicated cases and more severe mental illness Training of intern psychologists Referral support to primary healthcare level	Mental Health Care Act Hospital protocol
Medical officers	Assessment, diagnosis and medical treatments for mental disorders and other comorbid medical conditions for in-patients/out-patients Detoxification for individuals with substance misuse	Mental Health Care Act Hospital protocol Standard treatment guidelines and essential drug list for hospital level adults 2006
Psychiatrist (part time)	Assessment, diagnosis and psychiatric treatment for in-patients (emergency admissions, 72 h observation of involuntary patients, short-term acute in-patient care and upward referral to tertiary spec services Psychiatric referral service for confirmation and adjustment of diagnoses and treatment regimes for more complex psychiatric cases	Mental Health Care Act Hospital protocol Standard treatment guidelines and essential drug list for hospital level adults 2006
Psychiatric nurses	In-patient care/out-patient nursing care	Hospital protocol
Pharmacist	Ensure sufficient stock of medicine, package medicine for patients, distribute to primary healthcare facilities	Essential drug list Hospital protocol

Primary healthcare support team		
Primary healthcare manager	Supervision of primary healthcare clinics at subdistrict level	PRIME implementation toolkit including change management workshop
Subdistrict master trainer	Training and support of facility-based trainers in PC101+	PC101+ training materials
District/subdistrict chronic care/mental health coordinator	Coordinating chronic care in the primary healthcare facilities Training and support of primary healthcare nurses in the emergency management and ongoing psychopharmacological treatment of psychiatric patients Linking discharged psychiatric patients with follow-up care at local clinics Training of traditional healers, spiritual leaders, police in identification and referral of mental disorders	PRIME implementation toolkit including change management workshop Mental Health Care Act and Department of Health training materials Hospital protocol Collaborative care model for schizophrenia Existing Department of Health training protocol
Psychiatrist (part time)	Psychiatric referral service for confirmation and adjustment of diagnoses and treatment regimes for more complex psychiatric cases	Standard treatment guidelines and essential drug list for hospital level adults 2006
District/subdistrict psychologist/intern psychologist	Referral to psychological service for patients requiring more complex psychological treatment Training, supervision andsupportfor lay counsellors	Hospital protocol PRIME South Africa counselling training manual Prime South Africa counselling supervision guidelines for lay counsellors
Family physician	Training, supervision and support for primary healthcare doctors identification and management of mental disorders	PC101+

Primary healthcare facility tier		
Facility manager	Management of clinic services	PRIME South Africa implementation toolkit including change management workshop
Primary healthcare doctors	Diagnosis of mental disorders and other comorbid medical conditions Management of comorbid medical conditions Initiation and reassessment of psychotropic medication Referral of patients with moderate/severe depression to lay counsellors for the provision of manualised group/individual counselling for depression comorbid with chronic conditions Onward referral of complex and severe cases to district psychologist/out-patient services/ psychiatric hospital Brief screening and intervention for alcohol misuse Emergency management and referral of patients with acute psychiatric conditions to the district hospital	PC101+ Collaborative care model for depression using referral protocol and documentation Mini drug master plan 2011/12-2012/13 Clinic protocol Standard treatment guidelines and essential medicines list for primary healthcare 2008
PC101 facility trainer	Facility training and supervision of primary healthcare nurses in PC101 +	PC101+ training materials
Primary healthcare nurse	Emergency management and transfer of acute psychiatric conditions to the district hospital Ongoing symptom management of chronic psychiatric conditions – repeat medication Referral of chronic severe mental illness to the psychosocial rehabilitation groups Identification of mild depressive symptoms and provision of psychoeducation using the ’stressed/miserable patient’ page of PC101+ Identification of moderate/severe depression using PC101+ and referral to primary healthcare doctor and to lay counsellors for the provision of manualised group/individual counselling Brief screening and intervention for alcohol misuse (PC101+) and onward referral if necessary Record information on patients with mental disorders Management/referral of comorbid medical conditions	PC101+ Clinic protocol Referral protocols using collaborative care models for schizophrenia, depression and alcohol misuse Chronic care form and MHIS Standard treatment guidelines and essential medicines list for primary healthcare 2008
Lay counsellor	Facilitation of manualised individual and structured group-based counselling for depression Provides mental health promotion materials and talks Pre- and post-HCT counselling	PRIME South Africa counselling guidelines PRIME South Africa mental health literacy materials
Information officer	Capture, and manage mental health components of the information system	MHIS and clinic protocols District health information system
Community health worker (outreach team)	Identification and referral of people with mental disorders Psychoeducation on mental illness and stigma and discrimination Follow-up and adherence counselling for patients who default on their medication Mental health promotion Conduct household visits inclusive of mental healthcare Referral of MHCUs in need of facility (clinic or hospital) care	2nd phase Department of Health training manual for community health workers

Community tier		
Social workers	Assisting psychiatric patients in their applications for disability grants Training and supervision of auxiliary social workers in the delivery of community-based psychosocial rehabilitation	DSD protocol PRIME South Africa psychosocial rehabilitation training manual
Auxiliary social workers	Group-based psychosocial rehabilitation for MHCUs with severe chronic mental disorders	PRIME South Africa psychosocial rehabilitation guidelines
User groups	Psychoeducational campaigns to improve mental health literacy and reduce stigma and discriminations	Material from Federation for Mental Health
Non-governmental organisations		PRIME South Africa mental health literacy materials

MHIS, mental health information system; HCT, HIV counselling and testing; MHCU, mental healthcare user; DSD, Department of Social Development.

## Discussion

The advantages of integrating mental health into existing service delivery platforms include the opportunity for the provision of holistic care; reduction in stigma; and leverage of existing resources to promote efficiency and greater effectiveness of health interventions.^32^ Given synergies with chronic care, unlike other country plans in this supplement, South Africa is in the fortunate position of being able to scaffold off the introduction of ICDM, with chronic care embracing a collaborative patient-centred approach, central to integrated mental healthcare. We have structured our discussion using the framework suggested by Patel *et al* on how to integrate mental health into other healthcare platforms.^32^ This framework incorporates three aspects: assessment and customisation; tasks and human resources; and standardisation.

First, assessment and customisation includes active collaboration of service managers and providers to accurately assess what can feasibly be delivered by the platform, and what lies beyond the scope of the platform and needs to be referred. The participatory nature of the ToC and quality improvement workshops helped facilitate customisation of the MHCP but a number of issues that emerged from the pilot were overlooked.

These include first, that the collaborative care models adopted by the MHCP require a paradigm shift in the approach to care.^33^ Although ICDM provides a potentially enabling platform for integration of mental health, staff need to be orientated to this approach. Change management workshops to orientate facility managers and service providers to the ICDM and integrated mental health have thus been included in the revised MHCP at an organisational level.

Second, with respect to the increased burden of emotional labour that accompanies mental healthcare, to minimise burnout and assist service providers who may experience mental health difficulties of their own, the revised MHCP includes (a) strengthening the employee assistance programme: and (b) instilling more containing leadership including stress management (included in the change management workshops).

Third, in relation to role clarification, the human resource mix and associated skills sets and resources to achieve the plan are contained in Table 5. Notwithstanding evidence of the effective use of lay health workers in other low- and middle-income countries to increase access to psychosocial interventions,^34^ poor role clarification and marginalised status of existing lay counsellors in the South African healthcare system^35^ resulted in two major difficulties: a lack of confidence and reticence to take on additional counselling duties on their part; and low referrals by primary healthcare nurses who did not trust their competencies to counsel patients effectively. This has been addressed in the implementation toolkit of the revised MHCP through: (a) more clearly identifying a case manager (primary healthcare nurse) responsible for monitoring patient progress at primary healthcare level (which has been a need identified in the MHCP in India^36^ in this supplement); and (b) providing greater role clarification of lay counsellors.

A further difficulty has been harnessing human resources from other sectors, particularly auxiliary social workers from the Department of Social Development and NGO sector to facilitate psychosocial rehabilitation groups. Greater formal collaboration between the Department of Health and Department of Social Development at national and provincial level has been identified as a strategy to address this.

Standardisation refers to monitoring of patient progress to assess whether care needs to be adjusted or ‘stepped up’.^32^ This approach is commonly used in chronic care to monitor remission and obviate patients falling through the cracks. It has been incorporated into the collaborative care models for all three conditions but was not evaluated in the pilot study as no patient reached the point of needing upward referral.

In relation to the human resource requirements and costing of the PRIME South Africa MHCP, readers are referred to the cross-country costing paper in this supplement,^37^ where the number of additional full-time equivalent staff and associated costs for increasing coverage for the priority conditions in the MHCP are estimated. Given that mental health is part of ICDM in South Africa, the cost of increasing coverage of integrated mental healthcare in South Africa^37^ will have to be borne by the existing primary healthcare budget and thus have to compete with other chronic care priorities. For planners to see the value of integrated mental healthcare, the need for cost–benefit studies to show the cost savings and impact of integrated mental health on improved health outcomes in chronic care in South Africa emerged as a priority.

### Limitations

An obvious limitation is the lack of integration into a maternal and child health service delivery platform. This is of concern given the high rate of maternal depression in South Africa^38^ and the negative impact on child developmental outcomes.^39^ Reasons for this include the current focus of the Department of Health on the ICDM and that in South Africa, maternal and child health occurs through a different service delivery platform in primary healthcare and would require the development of a different MHCP. This remains a challenge for the future. A further limitation is the difficulty in reaching men with alcohol misuse. Alcohol use is more prevalent in men in South Africa^40^ and given they comprise a minority (approximately a third) of primary healthcare clinic attendees in the North West province, the need to engage other health service delivery platforms such as private healthcare provided on the mines is indicated. An additional limitation of the MHCP is that there was minimal engagement with traditional healers in the development of the plan. Given that a large number of people with mental disorders consult traditional healers in South Africa, engaging traditional healers in the collaborative care models is an important task as we move forward.

### Recommendations

The recent adoption of a national mental health policy framework that embraces decentralised care and task sharing, together with mental health gaining ground as a public health priority, bodes well for future scaling up of the integrated mental healthcare services in South Africa. Although the PRIME South Africa MHCP does not serve as a blueprint for scale up to other districts given the great variety in resources available across districts in South Africa, the approach and implementation tools developed should aid this process.

An important consideration in scale up is the additional time demands on an already burdened system, reflected as an increase in the number of full-time equivalent's required for scaling up integrated care in the costing of the plan.^37^ A recommendation would be to use the introduction of national health insurance in South Africa to leverage additional resources for mental healthcare. To strengthen this possibility, cost–benefit studies demonstrating the health benefits and cost savings of integrated mental health are needed. This is especially important, given the lack of a dedicated budget for mental health within ICDM and the need to compete with other priority conditions for resources.
